# Case report: perioperative management of caesarean section for a parturient with mitochondrial myopathy

**DOI:** 10.1186/s12871-017-0385-4

**Published:** 2017-07-12

**Authors:** Qiang Zheng, Penghui Wei, Jinfeng Zhou, Haipeng Zhou, Fucheng Ji, Wenxi Tang, Jianjun Li

**Affiliations:** Department of Anesthesiology, Qilu Hospital of Shandong University (Qingdao), No.758 Hefei Road, Qingdao, 266035 People’s Republic of China

**Keywords:** Mitochondrial myopathy, Caesarean section, Parturient

## Abstract

**Background:**

Mitochondrial myopathies represent a group of disorders caused by mitochondrial defects that disrupt energy production. Most patients have issues from infancy to early childhood. Pregnancy in women with mitochondrial myopathy is uncommon and the management for these parturients is full of challenges.

**Case presentation:**

A 36-year-old woman with mitochondrial myopathy was scheduled for caesarean section under a combined spinal-epidural anaesthesia and multi-model analgesia. The parturient was safe and the delivery was performed safely and smoothly, but there were some complications after surgery due to the complex condition of the patient. After consultation with and treatment from multiple disciplines, both the parturient and neonate were well and discharged.

**Conclusion:**

It is important that patients with mitochondrial diseases are comprehensively assessed and monitored perioperatively.

## Background

Mitochondrial myopathies comprise a heterogeneous group of neuromuscular disorders induced by dysfunction of the mitochondrial respiratory chain that disrupt energy production [1]. In this report, we describe a case of 36-year-old woman with mitochondrial myopathy for a selective caesarean section and discuss our experience on perioperative management.

## Case presentation

We report a 36-year-old Chinese parturient (height 162 cm, weight 57 kg) with mitochondrial myopathy who was scheduled for caesarean section at 38 weeks of gestation. She developed ocular muscle paralysis and exercise intolerance at age 12 and was diagnosed with mitochondrial myopathy at 29 years old. Muscle biopsy was examined and the results showed that her mitochondrial myopathy is chronic progressive external ophthalmoplegia (CPEO). Analysis of muscle mtDNA showed a common large deletion from nucleotides 8483–13,446. Her regular medications included a multivitamin, idebenone and coenzyme Q10. She could not continuously walk more than two flights of stairs. The examinations on admission revealed a blood pressure (BP) 111/69 mmHg with a heart rate of 75 bpm and respiratory rate of 17 bpm at rest. The muscle strength of her four limbs was level 4, and neurological examinations were normal. The electrocardiography (ECG) confirmed sinus rhythm, complete right bundle branch block, left anterior branch block and clockwise rotation. The echocardiography showed left ventricular expansion (mild), regurgitation of the mitral and tricuspid valves (mild) and a reduced left ventricular ejection fraction (LVEF: 50%). Arterial blood gas analysis at rest and other laboratory examinations were not obviously abnormal.

No sedative premedications were used, considering the unpredictable degree of sedation and respiratory depression. Routine monitoring, included ECG, BP, pulse oximetry (SpO_2_) and body temperature. One hundred percent oxygen was given via face mask on arrival to the operation room. The arterial catheter was also implemented to monitor the arterial blood pressure and facilitate blood samples for arterial blood gas, serum lactate and blood glucose levels during the operation. After venipuncture, a combined spinal-epidural anaesthesia at lumber vertebra 3–4 was administered with 0.5% ropivacaine 2.5 ml to achieve a T_6_ sensory block. The temperature of the operation room was adjusted to 26 °C before arrival of the patient. The intravenous infusion fluid was warmed through a heating equipment. Additionally, a heating blanket was used to prevent hypothermia. Arterial blood gas was repeatedly analysed during the operation. Morphine (1 mg) was injected through an epidural catheter after delivery of the newborn. The weight of the neonate was 3.65 kg and Apgar scores were 9 and 10 at one and five minutes after delivery, respectively. The intraoperative blood loss was 400 ml and the patient received 1000 ml of Ringer’s solution during surgery. The operation time was 1 h and the patient’s body temperature was maintained between 36.4 °C and 36.8 °C.Sufentanil intravenous patient controlled analgesia (PCIA) was given. In addition, bilateral transversus abdominis plane blocks were administered with 30 ml of 0.25% ropivacaine (15 ml each lateral). Both the mother and neonate did well and were returned to the public ward after surgery.

In the afternoon of the first postoperative day, the parturient suddenly developed dyspnoea and cyanosis of the lips because of the low temperature in the ward. After being warmed and receiving oxygen supply via face mask, the patient was gradually relieved from respiratory distress. However, in the evening of postoperative day 4, the patient had difficulty breathing once again and was not alleviated by inhaling oxygen via face mask. Her ECG confirmed supraventicular tachycardia with frequent ventricular premature contractions, complete right bundle branch block and left anterior branch block. Arterial blood gas analysis showed the artery oxygen pressure (PaO_2_) was 56 mmHg and carbon dioxide pressure (PaCO_2_) was 87 mmHg. The brain natriuretic peptide (BNP) level was 1234 pg/ml. Echocardiography revealed a LVEF of 43%, dilatation of the right heart (mild), and decreasing systolic function of the left ventricle. According to the patient’s clinical symptoms and examinations, we diagnosed her with IIrespiratory failure and cardiac insufficiency. Endotracheal intubation was immediately performed for mechanical ventilation, and the patient was transferred to the intensive care unit (ICU) for further treatment. Biphasic positive airway pressure (BiPAP) ventilation was used for respiratory support to maintain arterial oxygen saturation (SaO_2_) more than 95% and the fraction of inspiration O_2_ (FiO_2_) was 40–60%. The positive end expiratory pressure (PEEP) was set to 4–8 cm H_2_O to improve oxygenation and mitigate the harmful effects of mechanical ventilation. She also received intravenous phosphocreatine, potassium chloride, albumin and diuretics for improving nutrition and the internal environment. After a series of therapy in the ICU, the patient was extubated 5 days later and was transferred to the neurology department for subsequent treatment. The patient was safely discharged home on the 22nd postoperative day.

## Discussion

Mitochondria are the principal producers of energy and main source of cellular metabolism in mammals. A series of five enzyme complexes located in the inner mitochondrial membrane participate in the production of adenosine triphosphate by utilizing the products of the tricarboxylic acid cycle and other metabolic approaches [2]. Defects in mitochondrial metabolism lead to the dysfunction of tissues with high energy requirements (e.g., brain, heart, and muscle) and result in clinical manifestations, such as fatigue, muscle weakness and exercise intolerance [3]. Mitochondrial diseases are genetically and phenotypically a heterogeneous group with a variable clinical course and an estimated incidence of 1 per 5000 [4]. Patients may survive from infancy to early adulthood [5]. Mitochondrial myopathies, one type of mitochondrial disease, comprise a group of neuromuscular syndromes caused by genetic dysfunction that disrupt the production of energy and usually result in clinical disorders of the muscle and other tissues, requiring a high energy supply [6].

This parturient requires special perioperative attention. First, the general preoperative assessment is crucial. Patients with mitochondrial diseases often have preoperative problems, such as decreased cardiac function or respiratory reserve, conduction disorders and metabolic dysfunction [7]. In this case, the patient had high degrees of atrioventricular block, including a complete right bundle branch and left anterior branch block. Anaesthesiologists may need to prepare atropine, isoprenaline, or even a temporary pacemaker, to cope with possible bradycardia during surgery. It is also important to consult with the attending internist before surgery.

Furthermore, intraoperative management is crucial for this patient. In addition to routine monitoring, including electrocardiogram, blood pressure, pulse oximetry and body temperature, an arterial catheter should be administered to monitor arterial blood pressure and to facilitate blood samples for arterial blood gas, serum lactate and blood glucose levels. It is important to maintain a normal body temperature and avoid perioperative shivering. We used a fluid heating device and heating blanket to prevent hypothermia during the operation. Many patients with mitochondrial diseases cannot metabolize lactate, and therefore, lactated Ringer’s solution should be avoided; most anaesthesiologists prefer to use 0.9% normal saline or Ringer’s solution without lactate [8]. It is also wise to avoid hypoglycaemia or hyperglycaemia during surgery. Although some patients with mitochondrial disorders have undergone various anaesthetic techniques without obvious adverse outcomes, the optimal type of anaesthesia for these patients remains unknown [9]. The three reasons why we chose neuraxial anesthesia for this parturient are as follows: a controlled T_6_ sensory block avoids respiratory depression; the patient can be provided postoperative pain relief through the administration of epidural opioids; and it can avoid general anaesthetic and muscle relaxants, which may lead to postoperative residual muscle blockade and deteriorate respiratory compromise. Although there is no apparent evidence that patients with mitochondrial disorders are susceptible to malignant hyperthermia, it is necessary to avoid the administration of agents known to trigger MH, such as succinylcholine and volatile anaesthetics [10]. Bupivacaine is identified to suppress lipid-based respiration in the myocardial mitochondria through inhibiting acylcarnitine exchange in rats. Carnitine deficiency increases sensitivity to bupivocaine-induced asystole. Therefore, bupivacaine may not be a suitable choice for patients with mitochondrial disorders [11, 12]. We chose ropivacaine as the local agent for this parturient without any adverse effects and a multimodal analgesia for postoperative pain management by administering epidural morphine, nerve block and sufentanil PCIA. Both the parturient and the newborn were doing well, and they were sent to the public ward after surgery.

Finally, general postoperative monitoring and management are also vital for this type of parturient. In this case, the patient developed respiratory depression and cardiac insufficiency after surgery due to the severity of the disease. After consultations with and treatments from multiple departments, the patient improved and was discharged home on the 22nd postoperative day.

## Conclusions

In our case report, patients with mitochondrial disorders should be comprehensively assessed before surgery. Careful monitoring was needed for this patient more than for other patients during the entire perioperative period. Interestingly, this patient may benefit from multimodal analgesia for postoperative pain management.

## Abbreviations

BiPAP: Biphasic positive airway pressure
BNP: Brain natriuretic peptide
BP: Blood pressure
CPEO: Chronic progressive external ophthalmoplegia
ECG: Electrocardiography
FiO_2_: Fraction of inspiration O_2_
ICU: Intensive care unit
LVEF: Left ventricular ejection fraction
PaCO_2_: Carbon dioxide pressure
PaO_2_: Artery oxygen pressure
PCIA: Intravenous patient controlled analgesia
PEEP: Positive end expiratory pressure
SaO_2_: Arterial oxygen saturation
SpO_2_: Pulse oximetry

## References

[1] Spiegel R, Saada A, Halvardson J, Soiferman D, Shaag A, Edvardson S, Horovitz Y, Khayat M, Shalev SA, Feuk L, Elpeleg O (2014). Deleterious mutation in FDX1L gene is associated with a novel mitochondrial muscle myopathy. Eur J Hum Genet.

[2] Tucker EJ, Wanschers BF, Szklarczyk R, Mountford HS, Wijeyeratne XW, van den Brand MA, Leenders AM, Rodenburg RJ (2013). Mutations in the UQCC1-interacting protein, UQCC2, cause human complex III deficiency associated with perturbed cytochrome b protein expression. PLoS Genet.

[3] Scaini G, Rezin GT, Carvalho AF, Streck EL, Berk M, Quevedo J (2016). Mitochondrial dysfunction in bipolar disorder: evidence, pathophysiology and translational implications. Neurosci Biobehav Rev.

[4] Kohda M, Tokuzawa Y, Kishita Y, Nyuzuki H, Moriyama Y, Mizuno Y, Hirata T, Yatsuka Y, Yamashita-Sugahara Y (2016). A comprehensive genomic analysis reveals the genetic landscape of mitochondrial respiratory chain complex deficiencies. PLoS Genet.

[5] Vogel H (2001). Mitochondrial myopathies and the role of the pathologist in the molecular era. J Neuropathol Exp Neurol.

[6] Milone M, Wong LJ (2013). Diagnosis of mitochondrial myopathies. Mol Genet Metab.

[7] Florian A, Ludwig A, Stubbe-Dräger B, Boentert M, Young P, Waltenberger J, Rösch S, Sechtem U, Yilmaz A (2015). Characteristic cardiac phenotypes are detected by cardiovascular magnetic resonance in patients with different clinical phenotypes and genotypes of mitochondrial myopathy. J Cardiovasc Magn Reson.

[8] Gurrieri C, Kivela JE, Bojanić K, Gavrilova RH, Flick RP, Sprung J, Weingarten TN (2011). Anesthetic considerations in mitochondrial encephalomyopathy, lactic acidosis, and stroke-like episodes syndrome: a case series. Can J Anaesth.

[9] Muravchick S (2008). Clinical implications of mitochondrial disease. Adv Drug Deliv Rev.

[10] Lerman J (2011). Perioperative management of the paediatric patient with coexisting neuromuscular disease. Br J Anaesth.

[11] Wong GK, Crawford MW (2011). Carnitine deficiency increases susceptibility to bupivacaine-induced cardiotoxicity in rats. Anesth.

[12] Niezgoda J, Morgan PG (2013). Anesthetic considerations in patients with mitochondrial defects. Paediatr Anaesth.

